# Supplementary material for: A prediction modeling based on SNOT-22 score for endoscopic nasal septoplasty: a retrospective study
Source: PeerJ. 2020 Sep 11;8:e9890. doi: 10.7717/peerj.9890 (PMC7489239; doi:10.7717/peerj.9890)
Supplement: Supplemental Information 2 [file peerj-08-9890-s002.pdf]

您好！

本问卷是常规的术后随访及临床调研项目，旨在了解鼻中隔偏曲术前、术后生活质量相关的状况，以便让患者了解自己的健康状况，并给予患者以合理的建议，及为临床统计提供数据。您的答案无所谓对错。本问卷填答的资料**绝对保密**，所以请您根据自己的真实情况填答，非常感谢您的合作！

一、下面是一些基本信息及一般身体健康状况的问题

1 编号 姓名

2 您的性别 ☐男 ☐女

3 您的年龄_________岁

4 您的体重_________kg

5 您的身高_________cm

6 您术前是否吸烟？ ☐吸烟 ☐不吸烟

7 您术前是否喝酒？ ☐喝酒 ☐不喝酒

8 您术前是否患有高血压？ ☐有高血压 ☐无高血压

9 您术前是否患有糖尿病？ ☐有糖尿病 ☐无糖尿病

10 您术前是否曾患有以下疾病？(可多选)

☐卒中史 ☐心梗史 ☐恶性肿瘤病史 ☐心脏病史 ☐哮喘史

二、下面您将看到一些关于鼻部症状的信息，我们想了解关于鼻部症状对您的影响，以下答案不分对错，请根据您的实际情况做出选择。根据您目前发生的问题的严重程度和频率大小，请在每个问题后面的数字上，对准困扰程度打勾。

1. 需要擤鼻涕

☐无 ☐很轻 ☐轻 ☐中度 ☐重度 ☐极重度

2. 打喷嚏

☐无 ☐很轻 ☐轻 ☐中度 ☐重度 ☐极重度

3. 咳嗽

☐无 ☐很轻 ☐轻 ☐中度 ☐重度 ☐极重度

4. 流清鼻涕

☐无 ☐很轻 ☐轻 ☐中度 ☐重度 ☐极重度

5. 鼻涕倒流（咽喉）

☐无 ☐很轻 ☐轻 ☐中度 ☐重度 ☐极重度

6. 流脓鼻涕

☐无 ☐很轻 ☐轻 ☐中度 ☐重度 ☐极重度

7. 耳闷胀

☐无 ☐很轻 ☐轻 ☐中度 ☐重度 ☐极重度

8. 头昏

☐无　☐很轻　☐轻　☐中度　☐重度　☐极重度

9. 耳痛

☐无　☐很轻　☐轻　☐中度　☐重度　☐极重度

10. 头面部疼痛或压迫

☐无　☐很轻　☐轻　☐中度　☐重度　☐极重度

11. 难以入睡

☐无　☐很轻　☐轻　☐中度　☐重度　☐极重度

12. 半夜容易苏醒

☐无　☐很轻　☐轻　☐中度　☐重度　☐极重度

13. 夜间睡眠质量不好

☐无　☐很轻　☐轻　☐中度　☐重度　☐极重度

14. 睡醒后觉得累

☐无　☐很轻　☐轻　☐中度　☐重度　☐极重度

15. 疲倦

☐无　☐很轻　☐轻　☐中度　☐重度　☐极重度

16. 工作效率下降

☐无　☐很轻　☐轻　☐中度　☐重度　☐极重度

17. 注意力不集中

☐无　☐很轻　☐轻　☐中度　☐重度　☐极重度

18. 沮丧、焦躁、易怒

☐无　☐很轻　☐轻　☐中度　☐重度　☐极重度

19. 忧虑

☐无　☐很轻　☐轻　☐中度　☐重度　☐极重度

20. 感觉不安或难堪

☐无　☐很轻　☐轻　☐中度　☐重度　☐极重度

21. 鼻塞

☐无　☐很轻　☐轻　☐中度　☐重度　☐极重度

22. 嗅觉减退

☐无　☐很轻　☐轻　☐中度　☐重度　☐极重度

23. 鼻出血

☐很少发生　☐经常发生

24. 在季节交替或者某一季节时上述不适症状尤为显著

☐是　☐否

25. 家族中是否存在跟您症状相似或明确诊断为鼻中隔偏曲的亲人

☐有　☐无

26. 是否使用过鼻用激素药物

☐是　☐否

27. 曾经接受过鼻部手术

☐是　☐不是

28. 鼻部曾受伤导致骨折

☐有　☐无

Hello!

This questionnaire is a routine postoperative follow-up and clinical investigation project, aiming to understand the status of life quality before and after nasal septoplasty, so that patients can understand their own health status, get reasonable suggestions, and provide data for clinical statistics.There is no right or wrong answer.The materials filled in this questionnaire are **strictly confidential**, so please fill in the answers according to your real situation. Thank you very much for your cooperation!

Firstly, Here are some basic information and questions about general health

Q1 No. Name

Q2 Gender ☐Male ☐female

Q3 ages ________years

Q4 weight ________kg

Q5 height ________cm

Q6 Did you smoke before fracture? ☐Smoking ☐No Smoking

Q7 Did you drink alcohol before fracture? ☐Drink ☐No

Q8 Did you suffer from high blood pressure before surgery? ☐YES ☐ No hypertension

Q9 Did you suffer from diabetes before fracture? ☐ YES ☐No diabetes

Q10 Have you suffered from any of the following diseases prior to your fracture?
(You can choose more than one)
☐History of stroke ☐History of myocardial infarction ☐History of malignant tumor
☐History of heart disease ☐History of asthma

Secondly, you will see some information about nasal symptoms, we want to know about the impact of nasal symptoms on you.

1.Need To Blow Nose
□No problem □Very mild problem □Mild or slight probem □Moderate problem □Severe problem □Problem as bad as it can be

2.Sneezing
□No problem □Very mild problem □Mild or slight probem □Moderate problem □Severe problem □Problem as bad as it can be

3.Cough

□No problem □Very mild problem □Mild or slight probem □Moderate problem
□Severe problem □Problem as bad as it can be
4.Runny Nose
□No problem □Very mild problem □Mild or slight probem □Moderate problem
□Severe problem □Problem as bad as it can be
5.Post Nasal Discharge
□No problem □Very mild problem □Mild or slight probem □Moderate problem
□Severe problem □Problem as bad as it can be
6.Thick Nasal Discharge
□No problem □Very mild problem □Mild or slight probem □Moderate problem
□Severe problem □Problem as bad as it can be
7.Ear Fullness
□No problem □Very mild problem □Mild or slight probem □Moderate problem
□Severe problem □Problem as bad as it can be
8.Dizziness
□No problem □Very mild problem □Mild or slight probem □Moderate problem
□Severe problem □Problem as bad as it can be
9.Ear Pain/pressure
□No problem □Very mild problem □Mild or slight probem □Moderate problem
□Severe problem □Problem as bad as it can be
10.Facial Pain/pressure
□No problem □Very mild problem □Mild or slight probem □Moderate problem
□Severe problem □Problem as bad as it can be
11.Difficulty Falling Asleep
□No problem □Very mild problem □Mild or slight probem □Moderate problem
□Severe problem □Problem as bad as it can be
12.Waking Up At Night
□No problem □Very mild problem □Mild or slight probem □Moderate problem
□Severe problem □Problem as bad as it can be
13.Lack Of A Good Sleep
□No problem □Very mild problem □Mild or slight probem □Moderate problem
□Severe problem □Problem as bad as it can be
14.Waking Up Tired
□No problem □Very mild problem □Mild or slight probem □Moderate problem
□Severe problem □Problem as bad as it can be
15.Fatigue During The Day
□No problem □Very mild problem □Mild or slight probem □Moderate problem
□Severe problem □Problem as bad as it can be
16.Reduced Productivity
□No problem □Very mild problem □Mild or slight probem □Moderate problem
□Severe problem □Problem as bad as it can be
17.Reduced Concentration
□No problem □Very mild problem □Mild or slight probem □Moderate problem
□Severe problem □Problem as bad as it can be

18.Frustated/ Restless/ Irrtable

□No problem □Very mild problem □Mild or slight probem □Moderate problem □Severe problem □Problem as bad as it can be

19.Sad

□No problem □Very mild problem □Mild or slight probem □Moderate problem □Severe problem □Problem as bad as it can be

20.Embrrassed

□No problem □Very mild problem □Mild or slight probem □Moderate problem □Severe problem □Problem as bad as it can be

21.Blockage/Congestion Of Nose

□No problem □Very mild problem □Mild or slight probem □Moderate problem □Severe problem □Problem as bad as it can be

22.Sense Of Taste Or Smell

□No problem □Very mild problem □Mild or slight probem □Moderate problem □Severe problem □Problem as bad as it can be

23.Epistaxis

□Hardly □Often

24.The above symptoms are particularly noticeable during the alternations of seasons or during certain seasons

□No □Yes

25. Is there a family member who has symptoms similar to yours or has been diagnosed with septal deviation

□No □Yes

26.Intranasal Corticosteroids History

□No □Yes

27.Nasal Surgery History

□No □Yes

28.History Of Nasal Bone Fracture

□No □Yes
